# Targeting the Sonic Hedgehog-Gli1 Pathway as a Potential New Therapeutic Strategy for Myelodysplastic Syndromes

**DOI:** 10.1371/journal.pone.0136843

**Published:** 2015-08-28

**Authors:** Jixue Zou, Zhigang Zhou, Liping Wan, Yin Tong, Youwen Qin, Chun Wang, Kun Zhou

**Affiliations:** 1 Department of Hematology, Shanghai Jiaotong University Affiliated First People's Hospital, Shanghai, People’s Republic of China; 2 Department of Intensive Care Unit, Shanghai Jiaotong University Affiliated First People's Hospital, Shanghai, People’s Republic of China; Indiana University School of Medicine, UNITED STATES

## Abstract

The complex mechanistic array underlying the pathogenesis of myelodysplastic syndrome (MDS) is still unclear. Although dysregulations of different signaling pathways involved in MDS have been described, the identification of specific biomarkers and therapy targets remains an important task in order to establish novel therapeutic approaches. Here, we demonstrated that the Shh signaling pathway is active in MDS and correlated it with disease progression. Additionally, the knockdown of Gli1 significantly inhibited cell proliferation *in vitro* and *in vivo*. Gli1 silencing also induced apoptosis and G0/G1 phase arrest. Furthermore, Gli1 silencing enhanced the demethylating effect of 5-aza-2'-deoxycytidine on the p15 gene promoter and subsequently promoted its expression by inhibiting DNA methyltransferase 1(DNMT1). Our findings show that the Shh signaling pathway plays a role in the pathogenesis and disease progression of MDS, and proceeds by modulating DNA methylation. This pathway may prove to be a potential therapeutic target for enhancing the therapeutic effects of 5-azacytidine on malignant transformation of MDS.

## Introduction

Myelodysplastic syndromes (MDS) are comprised of a diverse group of hematopoietic neoplasms characterized by aberrant myeloid differentiation and ineffective hematopoiesis that exhibit clinically as progressive cytopenias and bear a substantial risk of progression to acute myeloid leukemia (AML) [1]. The biological and clinical heterogeneity of MDS renders its effective treatment particularly challenging. Moreover, a higher incidence of MDS in older patients, possibly afflicted with other comorbidities, may affect their ability to receive aggressive MDS therapies such as traditional cytotoxic chemotherapy or hematopoietic stem cell transplantation (HSCT). Thus, alternative therapies with reduced toxicity are needed to treat these conditions.

Epigenetic silencing mediated by hypermethylation of suppressor genes at promoter regions is a common pathogenic mechanism in both MDS and AML[2–9]. The application of demethylating agents such as azacitidine and decitabine has increased the overall survival (OS) of MDS and prolongs the time to AML transformation [10, 11]. DNA methylation, mediated by DNA methyltransferase (DNMT) family of enzymes, proceeds via covalent modification of cytosines in the promoter regions of the gene [12]. DNA methylation patterns are mainly established and maintained by the action of DNMT1, which preferentially methylates hemimethylated DNA strands [13, 14]. Although approximately 50–60% of first-line treated MDS patients show moderate response to hypomethylating agents [10], a majority of these patients gradually lose this response and can be offered only supportive care similar to most primary resistant patients. Therefore, uncovering the underlying pathogenic mechanisms of MDS for improvement of therapy is an urgent challenge.

Hedgehog (Hh) has been recently shown as a critical mediator in the pathogenesis of various human cancers, and the important role of Hh pathway in hematopoietic stem cell self-renewal and in maintenance of cancer stem cells in leukemia was demonstrated recently [15]. The three Hh proteins: Sonic Hh (Shh), Indian Hh (Ihh), and Desert Hh (Dhh), share a common signaling pathway. Protein patched homolog 1 (Ptch1) is known to repress smoothened (Smo) under unligated conditions and this inhibition is released when the ligand (Shh, Ihh, or Dhh) binds to Ptch-1. This allows Smo to initiate an intracellular signaling cascade mediated by transcription factors. The important positive regulator of Hh pathway, glioma-associated oncogene homolog 1 (Gli1), is also a transcriptional target of Hh [16]. Bai *et al*. [17] demonstrated the expression of Shh and Gli1 in hematological neoplasms such as myeloid leukemia and multiple myeloma. Bhardwaj *et al*. [18] showed Shh promoted expansion of primitive human hematopoietic cells by regulating the downstream bone morphogenetic protein signals. These studies suggest that the Shh signaling is important in hematological neoplasms, which calls for further investigations into the potential role of the Shh pathway in the pathogenesis of leukemia.

However, it is still unclear whether the Shh pathway plays a similar role in the progression of MDS and this study is aimed at deciphering this potential role. Here, we established that the Shh signaling pathway is active in primary MDS cells and correlates positively with the International Prognostic Scoring System (IPSS) risk types. Inhibition of Shh signaling was observed to downregulate the expression of DNMT1, and thereby supressed cell survival in MDS. Furthermore, silencing of Gli1 led to an enhanced demethylating effect of 5-Aza-2′-deoxycytidine on the promoter of the p15 gene and subsequently promoted p15 expression via DNMT1 inhibition. This observation may provide an attractive novel treatment strategy compared to traditional therapeutic intervention in MDS.

## Materials and Methods

### Sample collection

The study group included 23 adults with untreated MDS and 9 cases of acute myeloid leukemia associated with MDS (18–73 years, median age 56 years) at the time of initial diagnosis and before primary therapy, accessioned at the Shanghai Jiaotong University-affiliated Shanghai First People's Hospital (Shanghai, China) between March 2013 and November 2014 (Table 1). The diagnosis of MDS was based on the criteria laid out by the World Health Organization (WHO) [19]. Based on the international prognostic scoring system (IPSS) risk types [20], cases were classified as low-risk (n = 13), intermediate-risk (int; n = 5), and high-risk (n = 5) types. Bone marrow samples from 9 hematologically normal bone marrow transplant donors (20–43 years old) were obtained as controls. A previous or concomitant occurrence of cancer with any chronic treatment was considered as criteria for patient exclusion. The study was approved by the ethics committee of Shanghai Jiaotong University Affliated Shanghai First People’s Hospital. Written and informed consent was obtained from each patient before enrollment in the study.

**Table 1:** The characteristics of patients.

Characteristics	N (%)
Age (years)	32
< 65	22 (69)
≥65	10 (31)
Sex	32
Male	20 (62)
Female	12 (38)
WHO classification	32
RA/RARS	8 (25)
RCMD	7 (22)
RAEB-1	3 (9)
RAEB-2	5 (16)
AML associated with MDS	9 (28)
IPSS risk group	23
Low	13 (56)
Intermediate-1, 2	5 (22)
High	5 (22)

RA, refractory anemia; RARS, refractory anemia with ringed sideroblasts; RCMD, refractory cytopenia with multilineage dysplasia; RAEB, RA with excess blasts. MDS, myelodysplastic syndrome; AML, acute myelocytic leukemia; WHO, world health organization; IPSS, International Prognostic Scoring System.

### Isolation and culture of human bone marrow CD34^+^ cells

Mononuclear bone marrow cells obtained from patients and healthy donors were separated by Ficoll density gradient centrifugation. CD34^+^ cells were isolated using magnetic-activated cell sorting (MACS) Direct CD34 Progenitor Cell Isolation Kit (Miltenyi Biotec), according to the producer's protocol. The purity of cell separation was established by staining with anti-CD34 antibodies and quantification by fluorescence-activated cell sorting analysis. The purity of CD34^+^ cells was adjudged to be 95% based on flow cytometry results.

### Cell lines and reagents

The MUTZ-1 cell line was provided by the Institute of Hematology of Zhejiang University (Hangzhou, China) and was established from the peripheral blood of a 5-year-old girl with MDS French-American-British (FAB) subtype refractory anemia with excess of blasts (RAEB) and progressed quickly to an acute myeloid leukemia (FAB M2). Del (5q) and additional rearrangements involving chromosome 5 [der (15) t (5; 15)] were detected [21]. Cells were cultured in RPMI-1640 medium using 10% fetal bovine serum and 1% penicillin-streptomycin (Gibco, Life Technologies) at conditions of 37°C, 100% humidity, and 5% CO_2_.

Shh-N peptide dissolved in phosphate-buffered saline was purchased from R&D Systems, USA. 5-Aza-2′-deoxycytidine (5-aza-dC) dissolved in DMSO was obtained from Sigma-Aldrich, USA.

### Cell viability assay

Cell viability was evaluated using the Cell Count Kit-8 (CCK-8) (Beyotime biotechnology, China). Cells in logarithmic growth phase were collected and 100 μl cells per well (1 × 10^5^/ml) were seeded in triplicate into 96-well plates, which were then incubated for 48 h. Subsequently, 10 μl of CCK-8 was added to each well and the absorbance after 4 h at 450 nm was measured by a microplate reader (Bio-Rad).

### Flow cytometry

Apoptosis assays were performed using APC Annexin V Apoptosis Detection Kit with Propidium Iodide (PI) (Biolegend, San Diego, USA). Cells were labeled with Annexin V-APC and PI following the producer’s protocol, after which they were analyzed using a flow cytometer (BD FACScan). Annexin V-positive cells were classified as apoptotic. Cells were fixed in 70% ethanol overnight at 4°C for cell cycle analysis. Subsequently, RNase A was added for 20 min at room temperature. Cells were stained with 20 μg/ml PI (Biolegend) and examined by a flow cytometer (BD FACScan).

### Transfection of recombinant lentiviral vector

Self-prepared recombinant lentiviral vector, shGli1, and control lentiviral vector were co-transfected into the 293T packaging cell line. Supernatant was collected 48 and 72 h after infection to harvest the recombinant virus. Control lentiviral vector and lentiviral vector shGli1 were co-transfected into MUTZ-1 cells and the transfection rate was over 95% (S1 Fig). The degree of Gli1 knock down was determined by real-time reverse transcription-PCR (qRT-PCR) and western blot analysis. The coding strands for Gli1 shRNA1 and Gli1 shRNA2 were 5′-GCCTGAATCTGTGTATGAAAC-3′ and 5′-GCTCAGCTTGTGTGTAATTAT-3′, respectively. The oligonucleotide sequence of the negative control was 5′-TTCTCCGAACGTGTCACGT-3′.

### Real time RT-PCR

Total RNA was isolated from MUTZ-1 cells and bone marrow CD34^+^ cells of patients using Trizol reagent. RNA (1 μg) was further treated with an RNA Reverse Transcription kit (Applied Biosystems, Life Technologies, USA). PCR amplification of respective genes was performed using IQ SYBR Green Supermix (Bio-Rad laboratories) with gene-specific primers (Table 2) in a 20 μL total reaction using StepOnePlus Real-Time PCR System (Applied Biosystems) under the following conditions: 95°C for 10 min, followed by 40 cycles of 15 s at 95°C, and 10 min at 60°C. Each measurement was performed in triplicate. The levels of mRNA expression were normalized to the expression of β-actin (ACTB). Gene expression was calculated by using the 2^−ΔΔCT^ method.

**Table 2:** Primer sequences for real-time PCR.

Genes	Forward (5’–3’)	Reverse (5’ –3’)
Shh	CGGAGCGAGGAAGGGAAAG	TTGGGGATAAACTGCTTGTA
Gli1	GGCTGGACCAGCTACATCAAC	TGGTACCGGTGTGGGACAA
Smo	ATCTCCACAGGAGAGACTGGTTCGG	AAAGTGGGCCTTGGGAACATG
DNMT1	AACCAACACCCAAACAGAAACT	CTCCATCTTCGTCCTCGTCAG
P15	GATGCGTTCACTCCAATGTCT	CAGTCTTCAGGTTTTCCTTTCTG
β-actin	CGTGCTGCTGACCGAGG	GAAGGTCTCAAACATGATCTGGGT

### Western blotting

Briefly, protein concentrations were examined by the BCA protein assay (Beyotime). Proteins were separated and transferred to a polyvinylidene difluoride (PVDF) membrane. Membranes were blocked with phosphate-buffered saline (PBS) containing 5% bovine serum albumin (BSA) and 0.1% Tween-20 for 2h, and incubated overnight with primary antibodies at 4°C. They were then incubated with secondary antibodies at room temperature for 2h. An enhanced chemiluminescence ECL (Millipore) was used to detect the proteins. β—Actin was measured as an equal loading control.

### Methylation-specific PCR

Genomic DNA was extracted from MUTZ-1 cells using the QIAamp DNA mini kit (Qiagen, German) following the manufacturer’s instructions, and was subjected to bisulfite conversion using sodium bisulphite in EZ DNA Methylation-Gold kit (Zymo Research, USA). The methylation status of CpG islands in the p15 gene promoter was determined by methylation-specific PCR (MSP). The p15 primers used for unmethylated alleles were as follows: sense; 5′-GGTTTTAAGTTGTAGAAGGATGAT-3′, and antisense; 5′-ATTAACCATAAACTTAACAACACTC-3′. The p15 primers used for methylated alleles were as follows: sense; 5′-GTTTTAAGTCGTAGAAGGACGAC-3′, and antisense; 5′-TTAACCGTAAACTTAACGACACTC-3′. DNA was amplified under the following conditions: 95°C for 3 min, followed by 40 cycles at 94°C for 30 s, 54°C for 30 s, 72°C for 1 min, and a final extension step at 72°C for 7 min. Amplified products were resolved using 2% agarose gels and visualized after staining with ethidium bromide under ultraviolet light.

### Immunofluorescence

Cells were fixed with 4% paraformaldehyde in PBS at room temperature for 30 min followed by a 2X wash with PBS and later permeabilized using 0.25% Triton X‑100. Cells were then blocked in 10% BSA for 1h followed by overnight incubation with rabbit monoclonal anti-Shh (IgG1, 1:500, Santa Cruz Biotechnology) or anti-Gli1 (IgG, 1:500, Abcam) antibodies at 4°C. After three washes with PBS, the mixtures were then incubated in the dark for 1 h with Alexa Fluor 488-conjugated donkey anti-rabbit, IgG secondary antibodies (1:200, Invitrogen). After three additional washes, nuclei were stained with 4’, 6-diamidino-2-phenylindole (Invitrogen) for 5 min. Immunofluorescence images were obtained by a Leica TCS SP8 laser scanning confocal microscope (Leica, Mannheim, Germany).

### Tumor xenografts in nude mice

Four-week-old athymic male nude mice were maintained in airflow chambers under specific pathogen-free conditions. MUTZ-1 cells stably transfected with sh-Gli1 or sh-control vectors were separately suspended in PBS at a final concentration of 1 × 10^8^ cells/ml, following which 0.1 ml suspended cells were subcutaneously injected into a single side of the flank. Each group, consisting of 10 mice, was sacrificed by cervical dislocation under diethyl ether anesthesia at 21 days post injection, and tumors were dissected and weighed. All procedures involving mice were conducted in accordance with the guidelines laid down by Shanghai Jiaotong University Affiliated Shanghai First People’s Hospital Animal Care. All efforts were made to minimize animal suffering, to reduce the number of animals used and to utilize possible alternatives to in vivo techniques.

### Statistical analysis

Data presented represent the mean ± standard error. The Student's *t*-test was performed for comparisons between two groups. One-way analysis of variance (ANOVA) was used for multiple comparisons between groups. Bonferroni adjustment was used for the Post Hoc test. Two-way ANOVA with post hoc analysis (Bonferroni test) was performed to determine the effects of Gli1 knockdown and time (or agent doses) on cell viability. Spearman correlation analyses were used to confirm the relationship between different genes. Statistical analysis was conducted using SPSS 13.0. P < 0.05 was accepted as statistically significant.

## Results

### Overexpression of Gli1 shows an increased risk along with a poor AML prognosis

**Fig 1:**
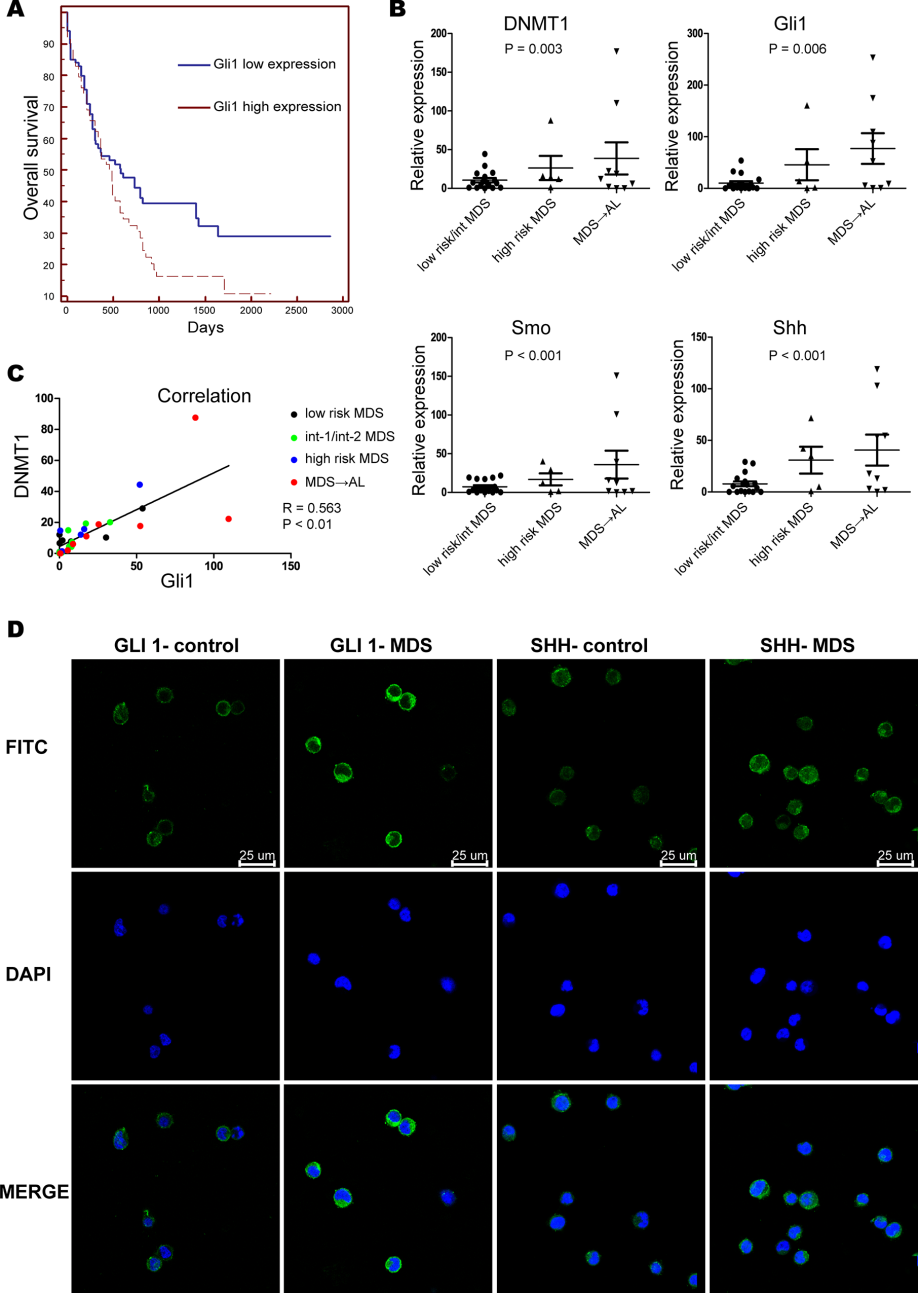
Gli1 predicts poor prognosis in AML and Shh-Gli1 signaling is activated in MDS. (A). Kaplan-Meier plots of overall survival (OS) in patients with AML, stratified by Gli1 expression. Data was obtained from the Cancer Genome Atlas (TCGA) database [23] (r = 1.41, P = 0.075). (B). Real-time PCR analysis of DNMT1, Gli1, Smo, and Shh in primary CD34^+^cells (n = 32) from MDS and acute myeloid leukemia associated with MDS marrow samples. (C). A statistically significant positive correlation of DNMT1 and Gli1 expression in MDS (Spearman’s correlation analysis, R = 0.563, P < 0.01). (D). Immunofluorescent staining of SHH and GLI1 in primary bone marrow-derived CD34^+^cells from MDS patients and control healthy donors.

In order to investigate the relationship between Gli1 and the clinical prognosis of AML, the prognostic value of Gli1 in the Cancer Genome Atlas (TCGA) database of AML, which includes data from 173 patients, was evaluated [22]. Results indicated an increased risk of Gli1 expression along with poor prognosis among AML patients (r = 1.41, P = 0.075) (Fig 1A). Since a large number of MDS patients eventually progress to AML, this finding indicates that the expression of Gli1 may be associated with the prognoses of MDS and AML.

### Activation of Shh–Gli1 signaling is associated with the IPSS risk types of human MDS

The gene expression of three essential components of the Shh pathway in CD34^+^ cells was investigated by quantitative real time PCR in order to ascertain whether Shh signaling is activated in MDS patients. To this end, 23 MDS and 9 acute myeloid leukemia associated with MDS patient samples were used. The expression of Shh, Gli1, and Smo was found to be significantly higher in high-risk MDS and acute myeloid leukemia associated with MDS patients in comparison with lower-risk MDS patients (Fig 1B). A strong positive correlation was observed between the expression levels of Shh, Gli1, Smo, and the IPSS risk types of MDS patients (P < 0.05).

Upon quantification, the expression level of DNMT1 in primary MDS CD34^+^ cells was found to be significantly increased in high-risk MDS CD34^+^ cells as compared with lower-risk MDS samples (Fig 1B). Moreover, a linear correlation was observed between DNMT1 and GLI1 mRNA levels (r = 0.563, P < 0.01) in MDS patients (Spearman’s correlation analysis) (Fig 1C). This indicated a probable participation of the Shh signaling pathway in MDS progression via DNMT1 modulation.

As expected, immunofluorescence assays indicated the presence of Shh and Gli1 proteins in CD34^+^ cells from high-risk MDS patients (Fig 1D). These results suggested active Shh signaling in MDS that might play an important role in disease progression.

### Knockdown of Gli1 induces apoptosis and cell cycle arrest of MUTZ-1

**Fig 2:**
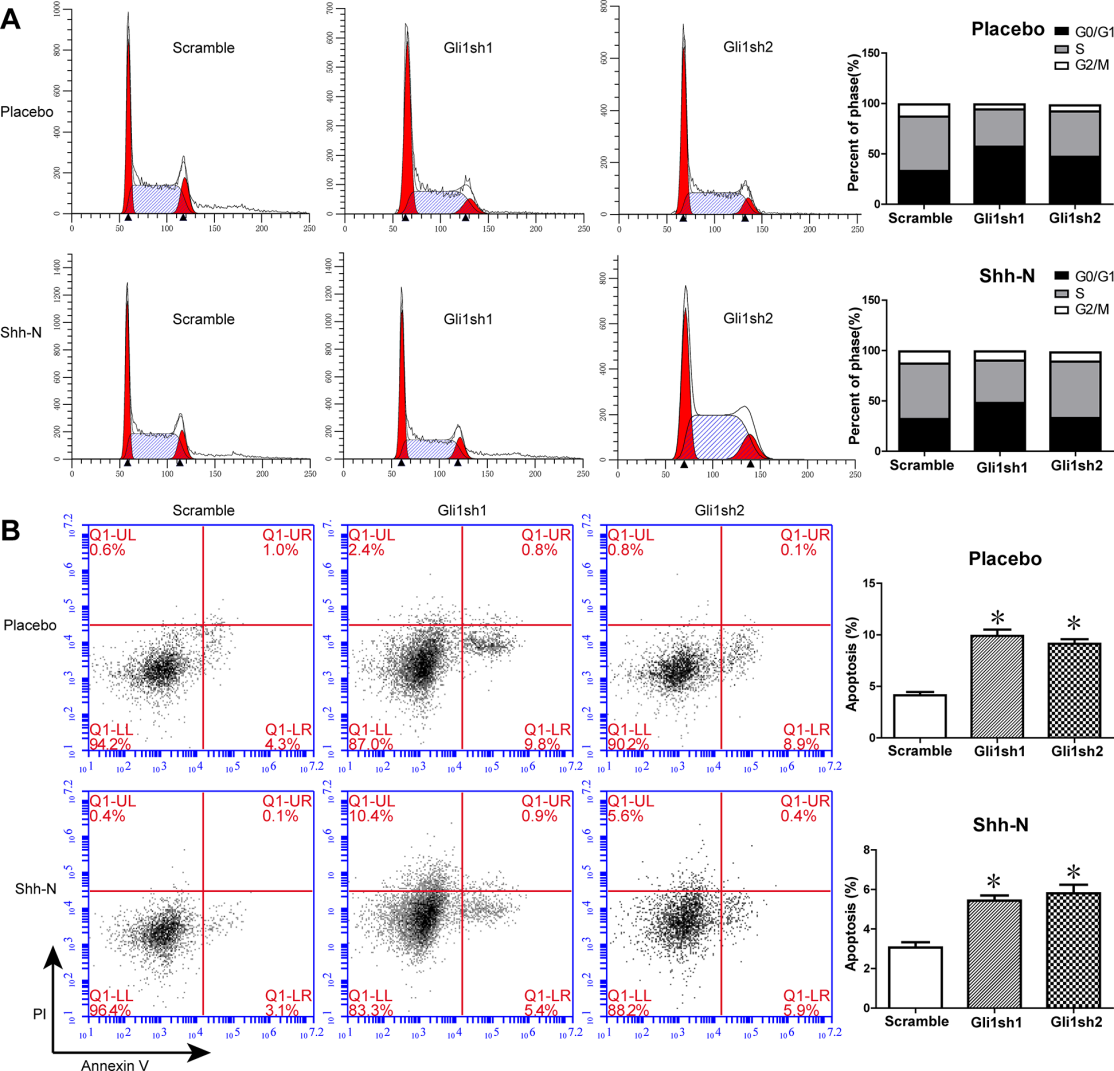
Knockdown of Gli1 induces MUTZ-1 cell apoptosis and cell cycle arrest. Gli1 knockdown MUTZ-1 cells were cultured in the presence (500 ng/ml) or absence of Shh-N for 48h. (A). Representative images of cell cycle distribution (left panel), and the percentage of cells in the G0/G1, S, or G2/M phases of cell cycle is indicated in the bar charts (right panel). (B). Representative images indicating cellular apoptosis determined by flow cytometry (left panel), and the sum percentage of early and late apoptotic cells indicated in the bar charts (right panel). All experiments were performed in triplicate and the results are expressed as average ± SEM. *P < 0.05.

The abnormal activation of Shh signaling in MDS cells led us to speculate its potential role in MDS progression. In this context, we silenced Gli1 expression in MUTZ-1 by shRNA in order to assess the role of Shh signaling in MDS cell survival. Further, we also examined the impact of modulating Shh signaling on cell cycle and apoptosis of MUTZ-1 cells by knockdown of *Gli1* using the exogenous human Shh peptide (Shh-N). A significantly increased proportion of cells in the G0/G1 phase was observed upon Gli1 knockdown as compared with the control group either with or without Shh-N (50% and 35% to 29%; 57% and 48% to 31%, P < 0.05) (Fig 2A). Further, the apoptotic rates of two MUTZ-1 cells transfected with Gli1 shRNA were also elevated (9.9% ± 0.6% and 9.1% ± 0.4%) in comparison with the control group (4.1% ± 0.3%) (Fig 2B). However, the apoptotic rates of two Gli1-silenced MUTZ-1 cells in the presence of Shh-N were significantly lower (5.4% ± 0.3% and 5.8% ± 0.4%, P < 0.05).

### Knockdown of Gli1 in MUTZ-1 cells decreases cell proliferation *in vitro* and *in vivo*

**Fig 3:**
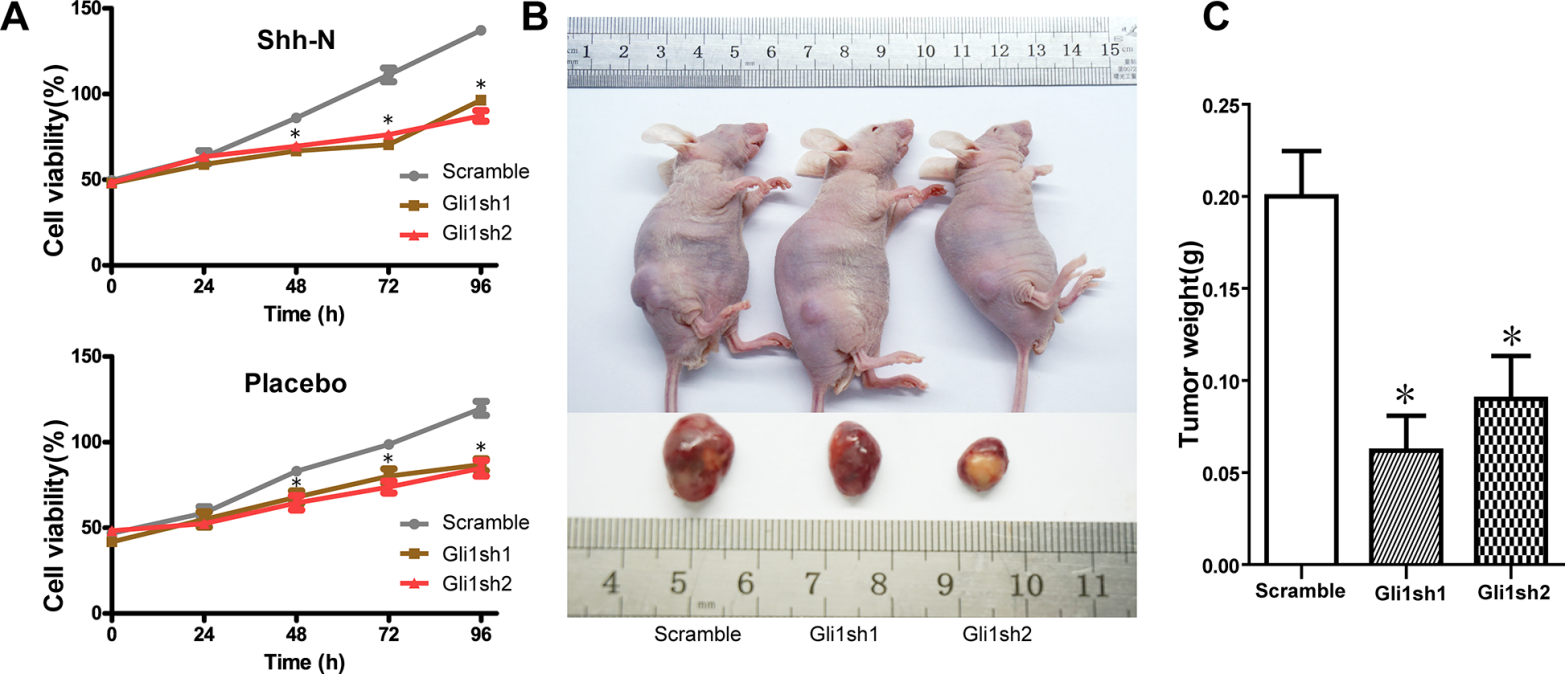
Knockdown of Gli1 inhibits *in vitro* and *in vivo* MDS cell growth. (A). CCK8 assays were performed to determine the proliferation of MUTZ-1 cells transfected with sh-Gli1 (Gli1 sh1 and Gli1 sh2) and control lentiviral vector (Scramble), wherein the knockdown of Gli1 was found to suppress MUTZ-1 growth *in vitro*. Two-way ANOVA with post hoc analysis (Bonferroni test) was performed to determine the effects of Gli1 knockdown and multiple time points on cell viability. (B). Representative images of tumor growth in nude mice. (C). Effects of Gli1 knockdown on tumor growth *in vivo*, wherein tumors developed from MUTZ-1 cells stably transfected with shGli1 showed smaller tumor volume and weights. *P < 0.05. Results are expressed as mean ± SEM.

Since the Shh signaling pathway led to an obvious induction of apoptosis and cell cycle arrest in MUTZ-1 cells, we determined whether this pathway also exerted any impact on cellular growth. CCK8 assays revealed that the knockdown of *GLI1* led to inhibition of MUTZ-1 proliferation in a time-dependent manner and effects of Gli1 knockdown with time were found to be statistically different between 48 and 96 hours (Fig 3A, P < 0.05). These results indicated an *in vitro* suppression of MUTZ-1 proliferation upon inhibition of Shh signaling.

To further determine whether the changes induced by Shh signaling in MUTZ-1 cells are also reflected *in vivo*, we investigated the tumorigenicity of MUTZ-1 cells transfected with Gli1 shRNA in athymic nude mice. Significant differences were observed in the tumor size and weight between Gli1 silenced and control groups (Fig 3B and 3C).

### Gli1 silencing enhances the demethylating effect of 5-aza-dC on p15 gene promoters and subsequently promotes p15 expression via DNMT1 inhibition

**Fig 4:**
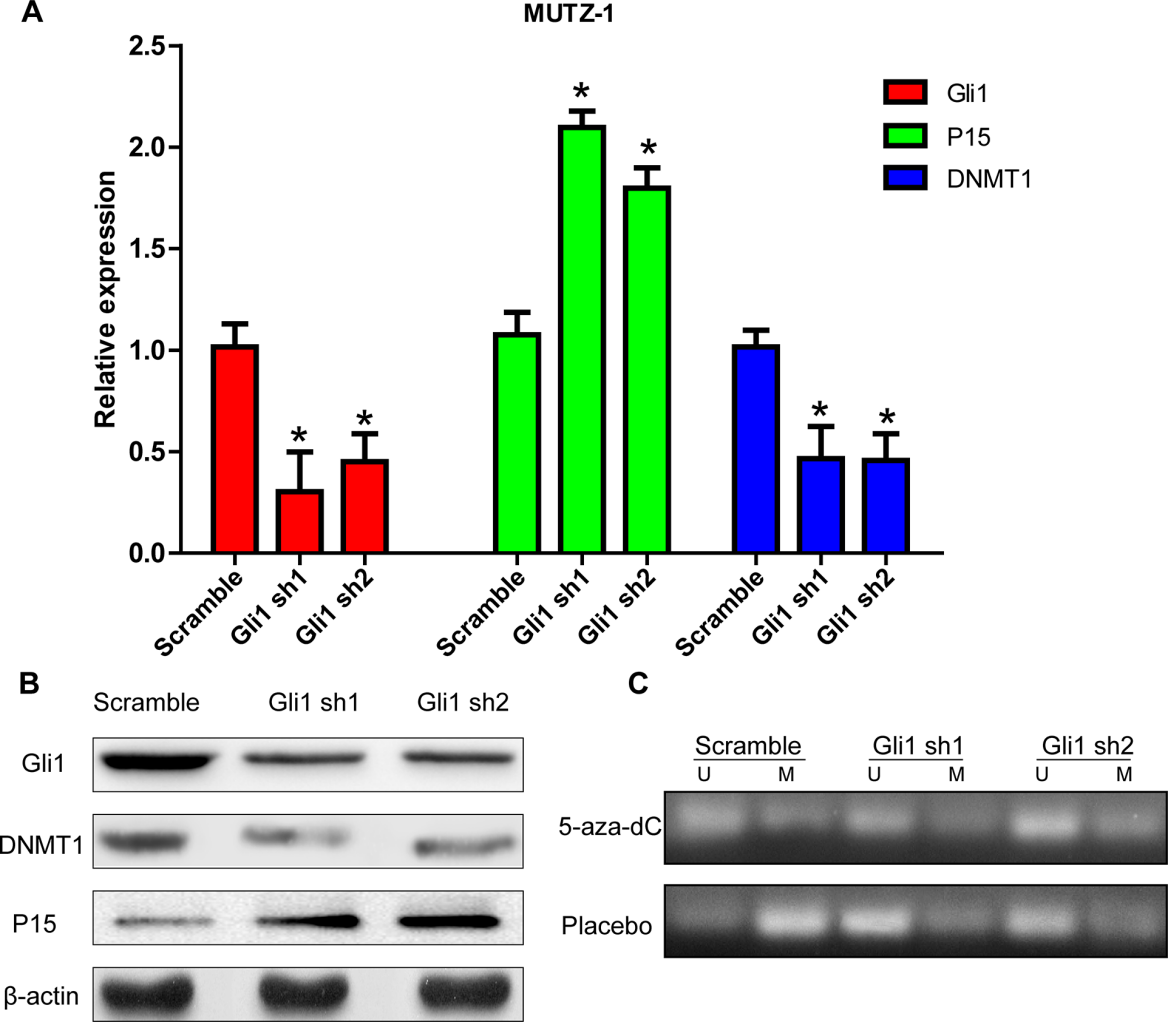
Gli1 silencing enhanced the demethylating effect of 5-aza-dC on p15 and subsequently promoted p15 expression. (A). Relative expression of DNMT1 and p15 mRNA upon Gli1 knockdown was analyzed by real-time PCR. Expression levels of beta-actin (ACTB), used as a housekeeping gene, were taken as control. Fold-change was calculated with the 2^−ΔΔCt^ method compared with controls. All experiments were performed in triplicate and the results are expressed as average ± SEM. *P < 0.05. (B). Western blot analysis of the effects of Gli1 knockdown on DNMT1 and p15 protein expression. Each sample was normalized to the respective related beta-actin (ACTB) expression. (C). Methylation-specific PCR for identification of changes in the methylation status of p15 promoter in MUTZ-1 cells transfected with sh-Gli1 (Gli1 sh1 and Gli1 sh2) and control lentiviral vector (Scramble) treated with or without 5-aza-dC (2 μM) for 48h. Images represent PCR-amplified products separated on 2% agarose gels and visualized under UV light after staining with ethidium bromide.

Next, we analyzed the changes in DNMT1 expression upon silencing of the important transcription factor Gli1, in order to identify the potential downstream pathways responsible for activation of Shh signaling in MUTZ-1 cells. Real-time PCR and western blot analyses confirmed that knockdown of Gli1 in MUTZ-1 cells led to significantly reduced DNMT1 expression both at the mRNA (Fig 4A) and protein (Fig 4B) levels. In addition, expression of the tumor suppressor gene p15 was significantly upregulated after blocking of the Shh pathway.

Since *Gli1* silencing led to increased p15 expression in MUTZ-1 cells, we performed methylation-specific PCR (MSP) to analyze whether its knockdown promoted p15 expression by influencing its methylation level. Compared with the placebo group, the methylation level of p15 with 5-aza-dC for 48h was obviously downregulated. These levels underwent a significant augmentation after knockdown Gli1 in MUTZ-1 cells (Fig 4C). These findings indicate that the Shh pathway may inhibit p15 by modulating its methylation level via DNMT1 overexpression.

### Gli1 silencing enhances the inhibitory effects of 5-aza-dC on MUTZ-1 cell growth

**Fig 5:**
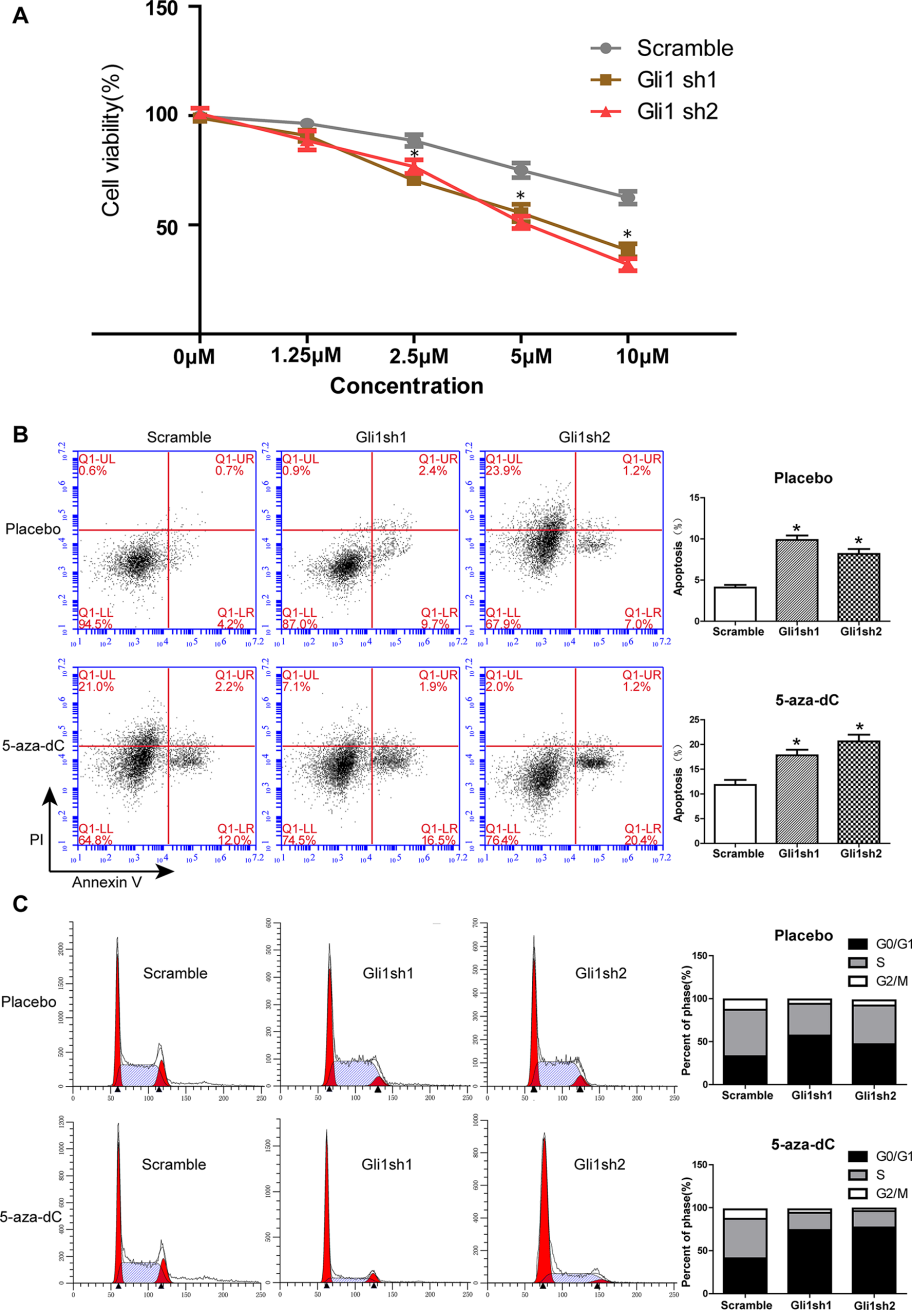
Gli1 silencing significantly sensitizes 5-aza-dC to inhibit MUTZ-1 cells. Gli1 silenced and scramble MUTZ-1 cells were cultured with or without 5-aza-dC (2 μM) for 48 h. (A). CCK8 assays were performed to determine the proliferation of MUTZ-1 cells transfected with sh-Gli1 (Gli1 sh1 and Gli1 sh2) and control lentiviral vector (Scramble) in the presence of different concentrations of 5-aza-dC. Two-way ANOVA with post hoc analysis (Bonferroni test) was performed to determine the effects of Gli1 knockdown and 5-aza-dC on cell viability. (B). Representative images of cell cycle distribution (left panel), and the percentage of cells in the G0/G1, S, or G2/M phases of the cell cycle are indicated in the bar charts (right panel). (C). Representative images of cellular apoptosis determined by flow cytometry analysis (left panel), and the sum percentage of early and late apoptotic cells indicated in the bar charts (right panel). *P < 0.05. Results are expressed as mean ± SEM.

To further investigate the effects of downregulation of the Shh signaling and demethylation on MDS cell survival, Gli1-silenced and scramble MUTZ-1 cells were cultured in the presence of 5-aza-dC, an inhibitor of DNA methyltransferase. Results of the CCK8 assay showed a 5-aza-dC dose-dependent inhibition of MUTZ-1 growth and effects of 5-aza-dC doses were found to be statistically different between 2.5μM and 10 μM (Fig 5A, P < 0.05). The Gli1 knockdown MUTZ-1 cells were more prone to inhibition compared with the scramble cells (P < 0.05). Proliferation of the two Gli1 knockdown MUTZ-1 cells was respectively 61.3% and 50.9% lower (38.3 ± 3.0%, 31.8 ± 2.8% to 62.4 ± 3.0%) than scramble cells when cultured in the presence of 10 μM 5-aza-dC for 48 h. Consistently, the effect of 5-aza-dC on MUTZ-1 cells was observed to increase upon shRNA Gli1 silencing (17.8% ± 1.1% and 20.6% ± 1.3% to 11.8% ± 1.0%, P < 0.05) (Fig 5B). 5-aza-dC-mediated inhibition of DNA methylation in combination with knockdown of Gli1 resulted in a synergistic induction of apoptosis of MUTZ-1 cells.

Flow cytometry was used to analyze cell cycle changes in order to investigate the mechanistic details of Gli1 knockdown on apoptosis of MUTZ-1 cells. Significantly higher number of cells were arrested in the G0/G1 phase after Gli1 knockdown combined with 5-aza-dC treatment as compared to those in the scramble group (74% and 77% to 41%, P < 0.05)(Fig 5C), suggesting synergistic interplay between Gli1 knockdown combined with 5-aza-dC treatment.

## Discussion

Dysregulation of the Shh pathway has been observed in tumorigenesis of human cancers including hematopoietic disorders [23]. Furthermore, inappropriate activation of this pathway has been confirmed in lymphomas and leukemias. Cellular differentiation failure is associated with the evolution of MDS into secondary AML, which renders the clinical management of MDS challenging. Here, we analyzed the status of the Shh-Gli1 signaling pathway in MDS, mainly since the mechanisms underlying the pathogenesis of MDS are still unclear. Activation of the Shh-Gli1 signaling pathway was found to display only insignificant differences between the IPSS low-risk and intermediate-risk types of MDS. However, the pathway was clearly activated in the high-risk MDS and acute myeloid leukemia associated with MDS samples. These findings indicate that the Shh-Gli1 pathway may be linked to a more progressive type of MDS, and this further raises the question whether a higher Shh-Gli1 expression may serve as a marker of poor prognosis. Indeed, analysis of the Cancer Genome Atlas (TCGA) database of AML that includes data from 173 patients showed an increased risk of Gli1 overexpression with poor prognosis among AML patients (r = 1.41, P = 0.075).

Our results are consistent with a previous study, which demonstrated the expression of Shh and Gli1 in both leukemic cell lines and primary leukemic blasts [24]. Shh was detected in 45% (9 out of 20) of the AML biopsies [17], and drug resistance was dramatically restored in the presence of hedgehog inhibitors in leukemic cells [24]. Flow cytometry analysis further confirmed that Gli1 could promote tumor growth by inducing cell cycle arrest at the G0/G1 phase and consequently decrease apoptosis. Further, Shh-Gli1 pathway blockage led to *in vitro* and *in vivo* suppression of MUTZ-1 cell proliferation. Consistent with our results, the Shh signaling has been demonstrated to be essential for the maintenance of myeloid leukemia cells [15]. Taken together, these findings suggest a participation of the Shh signaling pathway in the progression of MDS/AML. In our previous study, we had demonstrated that active Shh signaling from MDS bone marrow-derived stromal cells promoted the proliferation of MDS cell lines [25]. However, the precise molecular mechanism that elucidates the contribution of the dysregulated Shh signaling toward proliferation of MDS or leukemic blasts remains unclear.

In addition to genetic mutations, epigenetic changes are also predominantly involved in the pathogenesis of MDS/AML. In progressed MDS, the association between promoter hypermethylation and DNMT expression has been shown in all lineages [26]. In this study, increased DNMT1 expression was found in high-risk MDS cells. The association between DNMT and cancer is based on several observations that correlate the elevated DNMT expression and activity with occurence in several cancer types, wherein DNMT1 overexpression may lead to malignant transformation [27]. It is noteworthy that the expression of DNMT1 could be positively correlated with Gli1 in MDS and the knockdown of Gli1 in MUTZ-1 cells significantly reduced DNMT1 expression. A recent study has also reported on the observation of a substantially decreased DNMT1 expression after Gli1 interference in pancreatic cancer cells [28]. However, Gli1-mediated regulation of DNMT1 expression has not yet been reported in hematopoietic disorders. Our findings indicate that the Shh-Gli1 pathway promotes malignant transformation of MDS cells possibly by promoting DNA hypermethylation.

Olaf *et al*. showed that the aberrant methylation of p15 promoter was associated with increased DNMT1 expression in MDS. A strong association between p15 methylation and transformation to AML was also previously confirmed in MDS [4, 8, 29]. Hypermethylated p15 levels also mark the progression to a more aggressive phenotype accompanied with poor prognosis [30]. Given the limited therapeutic effects of demethylating agents used in MDS, some studies have focused on novel molecular targets coupled with traditional demethylation therapy, which may prompt therapeutic improvement [31]. To the best of our knowledge, we demonstrate for the first time that Gli1 silencing leads to enhanced demethylating effect of 5-aza-dC on the p15 promoter and subsequently promotes p15 expression by inhibiting DNMT1 in MUTZ-1 cells. This further confirmed that the downregulation of Shh-Gli1 signaling could lead to DNA demethylation and consequent p15 expression recovery in MDS.

We further discovered that a blockade of the Shh signaling pathway coupled with the demethylation agent, 5-Aza-2′-deoxycytidine, could synergistically inhibit MDS cell growth. Our results revealed that the Shh-Gli1 pathway may participate in the progression of MDS through independent but interrelated mechanisms based on abnormal DNA methylation. Several small molecules targeting the Shh-Gli1 pathway have already been identified [32]. Targeting these interactions, either individually or in combination, may provide an effective therapeutic alternative compared to conventional treatments.

In summary, the Shh signaling pathway is an important player in the pathogenesis and disease progression of MDS. Further, the downregulation of Shh-Gli1 signaling promotes the demethylating effect of 5-aza-2′-deoxycytidine on the p15 gene promoter by modulating DNMT1. Shh-Gli1 signaling blockade, individually or in combination with demethylating treatments, may provide a potentially novel therapeutic intervention for MDS.

## Supporting Information

S1 Fig The infection rate of MUTZ-1 cells. (TIF)

S1 Text ARRIVE Checklist. (PDF)
