# Supplementary figures and images for: Targeting the Sonic Hedgehog-Gli1 Pathway as a Potential New Therapeutic Strategy for Myelodysplastic Syndromes
Source: PLoS One. 2015 Aug 28;10(8):e0136843. doi: 10.1371/journal.pone.0136843 (PMC4552723; doi:10.1371/journal.pone.0136843)

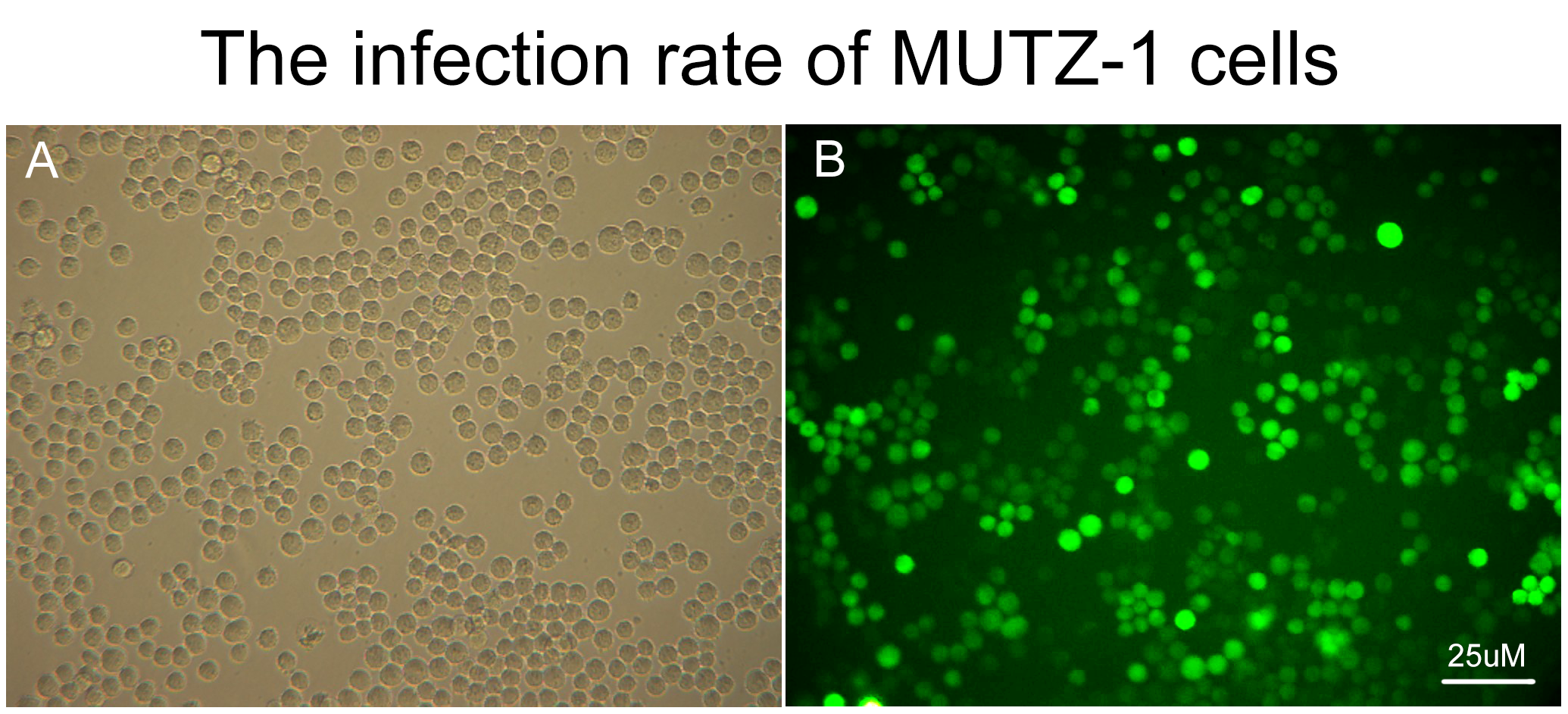

Supplement: S1 Fig — (TIF) [file pone.0136843.s001.tif]
